# The impact of clinical conditions and social factors on the psychological distress of cancer patients: an explorative study at a consultation and liaison service in a rural general hospital

**DOI:** 10.1186/1471-244X-13-226

**Published:** 2013-09-20

**Authors:** Juan Valdes-Stauber, Eva Vietz, Reinhold Kilian

**Affiliations:** 1 Zentrum für Psychiatrie Südwürttemberg, Department of Psychiatry and Psychotherapy I, University of Ulm, Ulm, Germany; 2 Outpatient Clinic, Bezirkskrankenhaus Kaufbeuren, Kaufbeuren, Germany; 3 Bezirkskrankenhaus Günzburg, Department of Psychiatry and Psychotherapy II, University of Ulm, Ulm, Germany; 4 Zentrum für Psychiatrie Südwürttemberg, Weingartshoferstraße 2, 88214, Ravensburg, Germany

**Keywords:** PO-Bado, Psycho-oncology, Psychological distress, Physical distress, Psychosocial support, Needs of cancer patients

## Abstract

**Background:**

In recent decades, increasing attention has been paid to the subjective dimension of cancer, especially to psychosocial screening procedures, major psychiatric disorders but also psychological and psychosocial distress, and finally to met needs of oncologic patients. This study aims first to describe cancer patients in a rural hospital attended by a psycho-oncological consultation-liaison team, second to assess predictors for psychological distress in cancer patients, and finally to identify predictors for recommendation of further psychosocial support.

**Methods:**

The sample (n = 290) comprises a full survey of patients at breast and bowel cancer services (n=209) and patients referred by other medical and surgical services because of psychosocial impairment (n = 81). All patients were assessed by means of the PO-Bado (Psycho-Oncological Basic Documentation) expert rating scale. Assessment of predictors for psychological distress was conducted by multivariate regression models and assessment for predictors for need for outpatient psychosocial support by a logistic regression analysis. All analyses were conducted using STATA 12.

**Results:**

Most members of the assessed sample (average age 65, 82% women) were not severely impaired from a functional and psychological point of view. A total of 14% had received psychiatric treatment before. Mood swings, anxiety, grief, and fatigue were the most important distress symptoms. Selectively referred patients vs. full survey patients of cancer centres, as well as bowel vs. breast cancer patients show a higher level of psychological and physical distress. Fatigue, assessed metastases, and functional limitations were the best predictors for psychological burden. Referral mode, gender, age, family problems, fatigue, and previous psychiatric treatment were associated with further need of psychosocial support.

**Conclusions:**

Psycho-oncological consultation and liaison services may offer support to patients in an early stage of cancer, especially in cancer centres. Because of selectively referred patients show a higher burden, the use of basic screening instruments could be meaningful. Fatigue, metastases status, and functional limitations may better predict psychological distress than pain, duration of illness, psychosocial conditions or previous psychiatric treatment. More attention has to be paid to outpatient follow-up with older cancer patients, those with family problems, and those suffering from significant fatigue.

## Background

In recent decades, increasing attention has been paid to the subjective dimension of cancer, especially to psychological and psychosocial distress. Usually, the concept of psychological distress includes major psychiatric disorders as well as minor psychological distress and psychosocial morbidity. The most frequent major psychiatric disorders are depression, anxiety, and adjustment disorders [1,2]. Mitchell et al. summarized in a meta-analysis of 94 interview-based studies the prevalence of psychiatric disorders in oncological, haematological, and palliative care settings: some combination of mood disorders occurred in 30–40% of patients in hospital settings without a significant difference between palliative and nonpalliative care settings [2].

Sufficient knowledge about psychosocial distress and mental disorders as well as screening procedures is urgent concern- to improve psychosocial care for cancer patients [3]. Two simple instruments measuring distress and mood were found to have acceptable levels of sensitivity and specificity in detecting psychosocial morbidity [4]. Further self-rating scales with broad performance are the Beck Depression Inventory (BDI) and Hospital Anxiety and Depression Scale (HADS).

Expert rating scales are an important complement to self-rating scales. Some subscales of the European Organisation for Research and Treatment of Cancer’ QIQ-C30 Questionnaire are important in the assessment of functional aspects of health-related quality of life [5], and the Palliative Performance Scale of the Victoria Hospice Society [6] can assess functional capacity and autonomy. The Questionnaire on Stress in Cancer Patients has been developed and psychometrically evaluated in Germany in order to assess psychological distress in a disease-specific manner [7].

Fear of progression seems to be the most important distress in cancer patients. The Fear of Progression Questionnaire comprising five factors was developed to assess this concern [8]. The Psychological Distress scale was recently developed in France as an adaptation of the National Comprehensive Cancer Network Distress Thermometer [9]. Mitchell et al. identified 63 studies involving 19 tools designed to help clinicians identify depression in cancer settings [10]. In clinical practice, all tools should form part of an integrated approach involving further follow-up, clinical assessment, and evidence-based therapy. Opinions of cancer clinicians regarding routine distress screening are mixed [11]. Key barriers seem to be lack of training and support, low acceptability, and failure to link treatment to the screening results [11], but also an unfavorable perception of screening [12].

Many psycho-oncological investigations assess prevalence of major psychiatric disorders and psychological distress, as well as of predictors for distress and indication for further psychosocial treatment. According to Zabora & Macmurray, over the past ten years many guidelines, recommendations, and standards have been developed on the basis of the concept of psychosocial screening [13]. This study aims to identify characteristics and group differences of cancer patients treated in a rural general hospital as well as meaningful predictors for psychological distress and for indication of further psychosocial support on the basis of data collected by means of the Psycho-Oncological Basic Documentation (PO-Bado), a reliable and valid expert rating scale that provides a structured format for focussed psycho-oncological assessment and intervention [14]. Recent results related to psychological distress and to psychosocial support need are considered in the Discussion and conclusions section.

### Objectives

**Figure 1:**
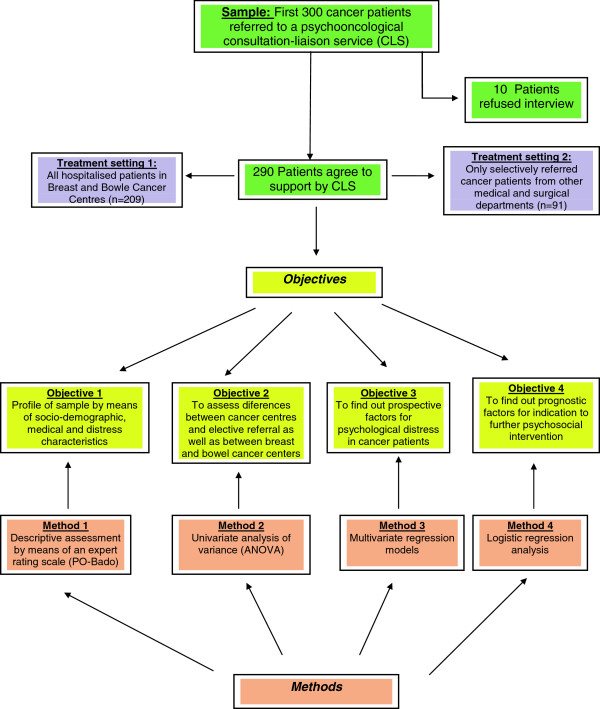
Rationale of the study.

The aims of this investigation are first, to describe cancer patients in a rural area in southern Germany (Kaufbeuren-Ostallgäu) attended by a psycho-oncological consultation-liaison service (CLS) on the basis of socio-demographic factors, care, distress, and functionality (assessed with performance status and functional limitations) variables by means of an expert rating scale; second, to assess differences between the group of patients belonging to cancer centres -full survey- and those electively referred because of suspected psychosocial comorbidity as well as between both breast and bowel cancer groups; third, to identify clinically relevant predictors of psychological distress rather than of major psychiatric disorders, and finally, to identify predictors of need for further outpatient psychosocial support after discharge (see Figure 1).

## Methods

### Setting description

The CLS under study started in 2009 and consists of a multidisciplinary team with two psychologists, one psychiatrist, one art therapist, and a supervising psychiatrist. This CLS is settled in the outpatient clinic of a district psychiatric hospital in the neighborhood of the general hospital under study. All interventions took place at the patient’s bedside per visit and were recorded in a clinical form. In addition, psychological and physical stresses as well as functional capacity were assessed and recorded by means of an expert rating scale (PO-Bado: Psycho-Oncological Basic-Documentation). Assessing clinicians were trained using the German version of the PO-Bado Manual of 2009 [15]. When managing difficulties arose (e.g. somnolence, nursing, diagnostic procedures, visit by relatives, etc.), the interview was completed later. If needed, patients were treated in psycho-oncological inpatient care (liaison service) or in outpatient care after discharge.

### Study sample

A total of 300 cancer patients referred to the CLS were included in this study, for which recruitment lasted 1.5 years, from summer 2009 to winter 2010. Ten of these patients refused contact with CLS. The remaining sample (n=290) was composed of two different populations: all newly admitted patients in the breast cancer and bowel cancer centres (n = 209), according to the recommendations of the German Cancer Society, and patients referred to CLS by medical and surgical departments because of suspected psychosocial comorbidity (n = 81) (see Figure 1). The total prevalence of major psychiatric disorders according ICD-10 was 6%, mainly mood and adjustment disorders. Psychiatric disorders were assessed by CLS therapists clinically -and not standardized- on the occasion of bedside consultation.

### Expert rating instrument

This study applied the PO-Bado, an expert rating scale developed in Germany by PO-Bado Work Groups in Munich and Heidelberg. PO-Bado has three versions: the standard version (PO-Bado, 17 items), the short form (PO-Bado SF, 7 items), and the breast cancer version (PO-Bado BC, 21 items) [16]. The standard version includes socio-demographic and clinical data as well as a physical distress scale (4 items), a psychological distress scale (8 items), and an additional distress scale (4 items) rated on the basis of a five-point Likert scale (0 = not at all; 1 = a little; 2 = moderately; 3 = considerably; 4 = very much). The five-point Eastern Cooperative Oncology Group scale was integrated into the PO-Bado booklet to assess the current level of functioning. The documentation form is normally filled out after the first interview with a patient and records the patient’s condition over the last 3 days [14]. The psychometric properties suggest that the PO-Bado is a reliable and valid tool for assessing and differentiating the distress of patients with cancer, as well as treatment-related changes in distress [17].

### Statistical analysis

Psychological and physical distress items were considered as interval-scaled variables. Cancer subtypes were included in both treatment settings (full survey of cancer centres and selectively referred cancer patients). Base outcome for metastases was “assessment in process, results yet unknown.” Differences between referral mode and between breast and bowel centres were assessed by means of univariate ANOVA; Bartlett’s test was considered in each one calculation.

Multivariate linear regression analyses were computed for the seven dimensions of psychological well-being and for a summation score of the seven dimensions in order to identify relevant clinical and socio-demographic predictors for each issue being assessed. Cancer subtypes were included in two referral modes; socio-demographic and clinical data were independent variables in the computed regression models. We considered standardized regression coefficient beta (0 < beta < 1) instead of b. Using Bonferroni correction for α, significance level for the whole multivariate table decreases from 0.05 to 0,006 and for each multivariate block from 0.05 to 0.004. We applied robust standard errors for the estimation of confidence intervals and significance tests of the regression coefficients. The sample size decreased from 290 to 273 because of missing data for some variables. Possible multicollinearity of independent variables was calculated by means of the variance inflation factor (VIF).

Possible predictors for a need for more intensive psychosocial support after discharge were assessed by means of logistic regression analysis on the basis of odds ratios (OR) (see Figure 1). All analyses were conducted with STATA 12.

### Ethics

This research was based on routine clinical records and was not a prospective trial. On this note, the study was not subject to formal approval by the local ethics committee. Only patients who agreed to contact with the CLS were assessed, and this assessment took place in a routine clinical context, according to German standards, like that for all other patients attended by CLS. The PO-Bado was completed according to German psycho-oncological standards as part of standard documentation. Our investigation has an exploratory character with regard to the profile of cancer patients treated and aims to test the feasibility of PO-Bado in a rural CLS setting. All statistical calculations were conducted anonymously by removing of names; an identification of individual patients was not possible.

## Results

Women made up 82% of the sample (n = 290). Breast cancer was the most frequent diagnosis (48%), followed by colon cancer (25%), gynaecological (10%) and digestive (7%) localisations; remaining 10% suffered from another cancer type. Patients in the mean were 65 years old and had been ill for 101 days (SD 327); 16% were experiencing a second cancer occurrence or relapse. A third of the individuals lived alone, and more than half were retired. At least 14% had had psychiatric treatment before onset of the cancer, and psychosocial support was indicated for almost a third. More than half reported a large degree of normal activity in everyday life. Economic and family-related stresses were low. Three-quarters had undergone a surgical intervention due to cancer, but only 10% had had chemotherapy and 8% radiotherapy, because intervention by CLS occurred at an early stage of the disease (see Table 1).

**Table 1:** Medical and psychosocial sample characteristics

	**N**	**Statistics for the whole sample**	**Differences breast cancer/bowel cancer**	**Differences cancer centres/ referred if needed**
			**F(p) r. X**^**2**^**(p)**	**F(p) r. X**^**2**^**(p)**
***Sociodemographic characteristics***				
**Gender:** proportion women	290	82%		
**Age**	273	M = 65; SD = 13.4	**6.56 (0.011)**	0.02 (0.902)
**Steady relationships:**	285	64%/ 36%	0.62 (0.735)	0.17 (0.916)
Couple/ single				
**Having children**	279	78%	0.04 (0.982)	0.90 (0.636)
**Working situation:**	290	23%/ 59%/ 17%	6.06 (0.300)	1.38 (0.926)
Employed/retired/unemployed				
***Medical characteristics***				
**Diagnosis:** Breast /bowel/gynaecological/ digestive/other	290	48%/ 25%/ 10%/ 7%/ 10%		
**Metastases:**	290	30%/ 19%/ 51%	**22.4 (<0.001)**	**21.86 (<0.001)**
Yes/no/not known				
**First diagnosis/**recidive/2nd cancer	279	84%/ 9%/ 7%	8.08 (0.089)	3.86 (0.425)
**Surgery**	290	75%	**18.2 (<0.001)**	**23.9 (<0.001)**
**Chemotherapy**	290	10%	**14.3 (<0.001)**	3.25 (0.071)
**Radiotherapy**	290	8%	**12.5 (<0.001)**	0.13 (0.720)
**Performance status (Levels 0–4)**	290	24%/ 33%/ 18%/ 18%/ 7%	**63.8 (<0.001)**	**24.25 (<0.001)**
		M =1.48; SD = 1.22		
**Duration of illness** (days)	290	M = 101; SD = 327	**5.1 (0.024)**	0.34 (0.314)
**Psychiatric/psychological treatment in past**	276	14%	2.53 (0.283)	0.96 (0.618)
**Current treatment with psychotropic drugs**	266	20%	5.2 (0.075)	**16.9 (<0.001)**
**Additional serious disease:** No/yes/no known	290	30%/ 36%/ 34%	4.02 (0.134)	2.43 (0.297)
***Psychological distress***				
**Sum score psychological distress**	290	M =7.5; SD = 5.65	**14.1 (<0.001)**	**34.52 (0.017)**
***Physical distress***				
**Sum score physical distress**	290	M =3.7; SD = 3.01	**5.26 (0.022)**	2.70 (0.100)
***Other distress factors***				
**Problems in family/ significant others**	290	17%	0.48 (0.488)	0.34 (0.581)
**Economic/work-related problems**	290	4.5%	0.63 (0.425)	0.08 (0.770)
**Additional stressful factors**	290	3.4%	0.11 (0.733)	0.04 (0.842)
**Stressors unrelated to the illness**	290	13%	1.24 (0.265)	1.71 (0.190)
***Indication***				
**Professional psychosocial support is indicated**	290	30%	0.02 (0.879)	**31.9 (<0.001)**

Descriptive statistics for continuous and interval-scaled variables (M = Mean; SD = Standard deviation) as well as for categorical variables (percentages). Variables are assigned to the categories according to PO-Bado. For continuous and interval-scaled variables: ANOVA, F (p-value); for dichotomous variables: χ^2^ (p-value). Bold figures: significant differences (p < 0.05). Categories of the variable “Performance status”: 0 = normal activity; 1 = Symptoms, but nearly full ambulatory; 2 = Some bed time, but needs to be in bed less than 50% of normal working hours; 3 = Needs to be in bed more than 50% of normal working hours; 4 = Permanently confined in bed.

Anxiety, grief, and mood swings were the psychological distress categories with the highest scores, each reported with a high distress level by about a fifth of the sample. Fatigue and restriction in daily activities were the physical distress categories with the highest scores. Pain was scored as low. For more than 40% of the sample, cognitive impairment, helplessness, shame, and pain were not mentioned at all (see Table 2).

**Table 2:** Psychological and physical distress in surveyed sample according PO-Bado

***Compared groups***	**Comparison full survey in centres/ selectively referral**						**Comparison breast/ bowel centres**					
***Distress items***	**Group**	**Degrees of severity (%)**			**Mean(SD)**	**ANOVA:F (p) R**^**2**^	**Group**	**Degrees of severity (%)**			**Mean(SD)**	**ANOVA:F (p)**
Sleep disturbances	To	30	59	11	1.12 (1.00)		Ce	32	60	8	1.02 (0.93)	
	Ce	32	60	8	1.02 (0.93)	**7.46 (0.007)**	Br	32	61	7	0.99 (0.89)	0.44 (0.507)
	Re	25	56	19	1.38 (1.14)	0.03	Bo	32	57	11	1.08 (0.99)	
Mood swings	To	22	59	19	1.37 (1.11)		Ce	25	62	13	1.15 (0.99)	
	Ce	25	62	13	1.15 (0.99)	**33.08 (<0.001)**	Br	22	67	11	1.13 (0.92)	0.11 (0.740)
	Re	14	52	34	1.95 (1.22)	0.10	Bo	30	54	16	1.18 (1.12)	
Cognitive impairment	To	66	28	6	0.56 (0.95)		Ce	70	26	3	0.45 (0.85)	
	Ce	70	26	3	0.45 (0.85)	**9.78 (0.002)**	Br	75	23	2	0.36 (0.74)	**4.26 (0.042)**
	Re	54	34	11	0.84 (1.11)	0.03	Bo	62	33	5	0.62 (1.02)	
Helplessness/ vulnerability	To	52	40	8	0.80 (1.01)		Ce	59	36	5	0.62 (0.89)	
	Ce	59	36	5	0.62 (0.89)	**26.7 (<0.001)**	Br	59	38	3	0.58 (0.84)	0.66 (0.417)
	Re	33	49	18	1.28 (1.15)	0.08	Bo	57	34	8	0.68 (0.98)	
Anxiety/worry/ tension	To	21	58	21	1.53 (1.20)		Ce	23	62	16	1.33 (1.11)	
	Ce	23	62	16	1.33 (1.11)	**22.9 (<0.001)**	Br	21	64	15	1.30 (1.06)	0.24 (0.624)
	Re	15	47	38	2.06 (1.27)	0.03	Bo	26	57	17	0.69 (0.98)	
Shame/loss of esteem	To	78	20	2	0.27 (0.59)		Ce	82	17	0.5	0.20 (0.50)	
	Ce	82	17	0.5	0.20 (0.50)	**9.7 (0.002)**	Br	82	18	0	0.20 (0.45)	0.001 (0.971)
	Re	67	30	3	0.44 (0.76)	0.03	Bo	84	15	1	0.21 (0.58)	
Depression/grief	To	28	51	20	1.40 (1.22)		Ce	33	54	13	1.15 (1.10)	
	Ce	33	54	13	1.15 (1.10)	**35.2 (<0.001)**	Br	33	55	12	1.12 (1.06)	0.54 (0.463)
	Re	15	55	39	2.06 (1.30)	0.11	Bo	33	52	15	1.23 (1.17)	
Fatigue	To	21	67	12	1.24 (0.95)		Ce	25	66	9	1.09 (0.87)	
	Ce	25	66	9	1.09 (0.87)	**20.8 (<0.001)**	Br	28	66	6	0.97 (0.81)	**7.59 (0.006)**
	Re	11	68	20	1.64 (1.04)	0.07	Bo	19	66	15	1.32 (0.95)	
Pain	To	40	54	6	0.82 (0.88)		Ce	45	53	2	0.70 (0.77)	
	Ce	45	53	2	0.70 (0.77)	**17.3 (<0.001)**	Br	46	53	1	0.65 (0.69)	1.81 (0.180)
	Re	27	58	15	1.16 (1.04)	0.06	Bo	42	53	4	0.79 (0.89)	
Functional limitations	To	37	48	15	1.13 (1.15)		Ce	46	46	9	0.86 (1.01)	
	Ce	46	46	9	0.86 (1.01)	**52.3 (<0.001)**	Br	56	40	4	0.63 (0.87)	**22.3 (<0.001)**
	Re	15	54	31	1.87 (1.20)	0.15	Bo	26	57	17	1.29 (1.11)	

Groups: To = Total sample; Ce = Cancer centres; Re = selectively referred patients; Br = Breast centre; Bo = Bowel centre. ANOVA = Univariate analysis of variance. Mean and Standard deviation (SD) were computed on the basis of interval-scaled variables considered as continuous variables. p = Level of significance (0.05). F = Value in F- distribution. Differences between groups were calculated on the basis of ANOVA, F (p-value); Bartlett’s correction was considered. R^2^ = Share of explained variance (in order to compare with results of multivariate models in Table 3). Bold figures: significant differences (p < 0.05). Five-point Likert scale: 0 = not at all; 1 = a little; 2 = moderately; 3 = considerably; 4 = very much were converted in degrees of severity: not at all; a little + moderately = Low; considerably + very much = High.

Patients selectively referred because of suspected psychosocial comorbidity underwent less frequently surgical interventions, but showed more often confirmed metastases and a lower level of performance; they were more psychologically distressed, underwent more often a psychopharmacological treatment, indication for further psychological support was more frequently (see Table 1) and each one somatic as well as psychological strain was significantly higher (see Table 2) than for the group of full survey cancer centres. On the other hand, within the group of cancer centres, patients suffering from bowel cancer are significant older and duration of cancer disease was longer; they show a higher level of physical as well as psychological distress at all, were more often treated with chemotherapy and radiotherapy, metastases status was less frequently known at assessment time and surgical interventions were less frequent than for patients suffering from breast cancer (see Table 1); with regard to each one assessed strain, fatigue, restriction in daily activities, and cognitive impairment were significantly more frequent for the bowel cancer group (see Table 2). These differences disappeared in multivariate models, when important covariates were considered; otherwise, their explained variance increased in the multivariate models about a factor between 3 and 10 (see Table 3).

**Table 3:** Associations between socio-demographic and clinical variables with psychological distress

***Dependent variables***	**Mood swings**		**Cognitive impairment**		**Anxiety**		**Grief**		**Sleep disorders**		**Helplessness**		**Shame**		**Sum score psychological distress**	
***Regressors***	**BETA**	**p**	**BETA**	**p**	**BETA**	**p**	**BETA**	**p**	**BETA**	**p**	**BETA**	**p**	**BETA**	**p**	**BETA**	**p**
1. Referral mode	**0.146**	**0.017**	0.032	0.651	0.099	0.105	0.107	0.056	0.044	0.499	0.109	0.098	0.085	0.317	0.107	0.062
2. Age	−0.121	0.107	0.450	0.533	−0.098	0.227	−0.099	0.166	0.005	0.951	**−0.151**	**0.040**	−0.113	0.252	−0.129	0.051
3. Gender	0.106	0.075	−0.024	0.666	0.093	0.125	0.049	0.331	0.091	0.108	0.058	0.285	0.025	0.640	0.073	0.166
4. Steady relationships	−0.035	0.541	**0.138**	**0.023**	−0.041	0.475	−0.036	0.511	−0.023	0.705	−0.019	0.760	0.041	0.661	0.024	0.635
5. Working situation	0.035	0.653	−0.131	0.068	−0.047	0.554	−0.026	0.715	−0.017	0.832	0.079	0.285	−0.014	0.859	−0.041	0.565
6. Having children	−0.004	0.935	0.030	0.598	−0.030	0.540	−0.052	0.291	−0.085	0.104	−0.021	0.648	−0.002	0.968	−0.056	0.196
7. Problems in family	−0.004	0.938	−0.021	0.711	−0.079	0.117	**0.124**	**0.011**	0.070	0.226	0.007	0.909	−0.063	0.344	−0.069	0.110
8. Fatigue	**0.202**	**0.001**	**0.230**	**0.000**	**0.212**	**0.001**	**0.349**	**0.000**	**0.276**	**0.000**	**0.178**	**0.008**	**0.156**	**0.010**	**0.299**	**0.000**
9. Pain	**0.145**	**0.022**	0.041	0.578	**0.066**	**0.003**	0.060	0,290	0.099	0.191	0.061	0.350	0.079	0.398	0.081	0.162
10. No metastases	**−0.273**	**0.000**	−0.023	0.716	**−0.187**	**0.002**	**−0.155**	**0.007**	0.051	0.429	**−0.135**	**0.048**	0.086	0.216	**−0.042**	**0.007**
11. Metastases status unknown	**−0.165**	**0.021**	−0.011	0.878	−0.223	0.338	**−0.167**	**0.011**	−0.026	0.730	−0.115	0.139	0.108	0.165	−0.146	0.030
12. Duration of illness	−0.072	0.181	0.167	0.076	−0.054	0.177	−0.001	0.995	0.069	0.249	−0.007	0.902	−0.046	0.394	0.036	0.540
13. Previous psych. treatment	−0.009	0.864	−0.002	0.980	−0.070	0.658	−0.065	0.203	0.020	0.751	−0.042	0.482	−0.001	0.984	−0.053	0.273
14. Performance status	−0.036	0.642	0.449	0.493	−0.031	0.658	0.061	0.295	0.015	0.861	−0.001	0.992	−0.007	0.934	0.018	0.765
15. Functional limitations	0.122	0.146	**0.191**	**0.041**	0.163	0.053	0.146	0.056	0.040	0.692	**0.265**	**0.001**	0.195	0.066	**0.273**	**0.005**
***Constant***	1.63	0.000	0.04	0.914	2.24	0.000	1.87	0.001	0.26	0.522	0.95	0.007	0.27	0.255	9.23	0.000
***F/Prob > F***	11.66/ <0.001		4.01/ <0.001		10.41/ <0.001		20.39/ <0.001		3.23/ <0.001		7.30/ <0.001		2.70/ <0.001		19.02/ <0.001	
***R2***	0.34		0.27		0.32		0.46		0.16		0.31		0.14		0.45	
***N***	273		273		273		273		273		273		273		273	

b = Regression coefficient; p = Level of significance; R^2^ = Share of explained variance; F = value in F-Distribution. Base-outcome for metastases = confirmed metastases. Referral mode: 0 = Patients from Breast and Bowel centres; 1 = Patients referred by other services if mental status requires it. *Continuous variables*: 2, 12, and dependent variable “sum score psychological distress”; *Interval-scaled variables:* 8,9,14,15 and all dependent variables with the exception of Sum score psychological distress; *dichotomous variables:* Dep. Variables 1 (0 = breast and bowel centres; 1 = Referred if needed), 3 (0 = men; 1 = women), 4,5,6,7,13 (0 = no; 1 = given). Other dichotomous variables: not applicable (0), applicable (1). Bold figures: significant differences (p < 0.05); note: under Bonferroni correction p-level downsizes to 0.004.

**Figure 2:**
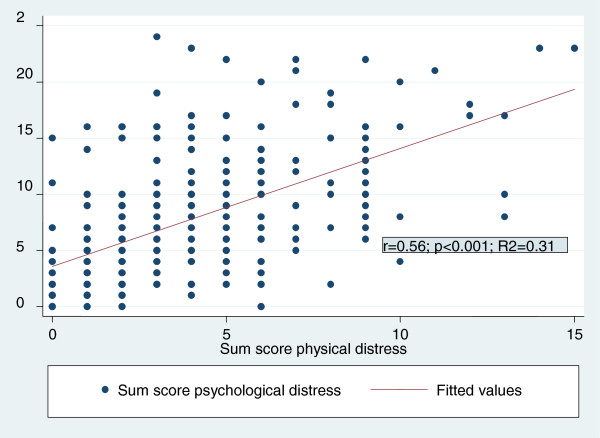
Association between physical and psychological distress.

We explored possible associations between physical and psychological distress by means of bivariate regression analysis. The scatter plot in Figure 2 demonstrates, on the basis of total distress scores, that increasing physical distress was positively associated with increasing psychological distress (b = 1.05; p < 0.001; Beta = 0.56). This effect disappeared when control variables were added (see Table 3). We did not find multicollinearity within independent variables by means of the variance inflation factor (VIF): mean VIF amounted 2.03, still under the threshold of 10. Three models showed a relatively high degree of explanation of variance: those for mood swings (34%), depression and grief (46%), and sum score of psychosocial distress (45%).

Differences between the group of patients attended in cancer centres and the group of selectively referred patients when psychosocial comorbidity was suspected found in ANOVA tests disappeared in multivariate models, especially under consideration of Bonferroni correction. Living in couple was negatively associated with cognitive impairment (p = 0.023) and older age with helplessness respectively vulnerability (p = 0.022), whereas pain was positively associated with mood swings (p = 0.022) and problems in family with grief (p = 0.011). Fatigue, assessed metastases, and functional limitations were the best predictors for psychological burden at all as well as for the majority of each one psychological distress item, even under consideration of Bonferroni correction (see Table 3).

Finally, we tried, by means of logistic regression analysis, to identify possible predictors for recommendation of further psychosocial support (see Table 4). The CLS team suggested outpatient support more frequently for women (OR = 3.76; p = 0.018), for treatment setting (selective referral vs. full survey) (OR = 3.13; p = 0.010), and for patients reporting fatigue (OR = 1.80; p = 0.038) or family problems (OR = 2.73; p = 0.026). For patients who had been in psychiatric or psychological treatment before admission, advice for further psychosocial treatment was notably more frequent (OR = 2.85; p = 0.026), but this advice decreased with age at a rate of about 4% per year (OR = 0.96; p = 0.037).

**Table 4:** Logistic regression analysis to assess prognostic factors for indication of further psychosocial support

***Dep.variable: Further psychosocial support is indicated***			
***Regressors***	**OR**	**p**	**95% CI**
**1. Referral mode**	**3.13**	**0.010**	**1.32; 7.42**
**2. Age**	**0.96**	**0.037**	**0.93; 0.99**
**3. Gender**	**3.76**	**0.018**	**1.25; 11.27**
**4. Steady relationships**	0.62	0.289	0.26; 1.48
**5. Working situation**	0.94	0.913	0.35; 2.58
**6. Having children**	1.13	0.779	0.46; 2.78
**7. Problems in family**	**2.73**	**0.026**	**1.13; 6.63**
**8. Fatigue**	**1.80**	**0.038**	**1.03; 3.14**
**9. Pain**	0.78	0.411	0.44; 1.39
**10. Sum score physical distress**	1.38	0.068	0.97; 1.92
**11. No metastases**	0.34	0.063	0.11; 1.06
**12. Metastases status unknown**	1.56	0.305	0.66; 3.65
**13. Duration of illness**	1.00	0,434	0.99; 1.00
**14. Prev. psychiatric. treatment**	**2.85**	**0.026**	**1.13; 7.16**
**15. Performance status**	0.64	0.062	0.40; 1.02
**16. Functional limitations**	1.28	0.463	0.66; 2.50
***Constant***	0.24	0.365	0.0; 5.24
***LR/Prob > Chi***^***2***^	115.54/<0.001		
***Pseudo R2***	0.37		
***N***	250		

OR = Odds ratio for dichotomous dependent variable; p = Level of significance; 95% CI: Interval of confidence for odds ratio; LR: Likehood ratio; R^2^ = Share of explained variance. Base outcome for metastases = Metastases confirmed. Referral mode: 0 = Patients from Breast and Bowel Centres; 1 = Patients referred by other services if mental status requires it. *Continuous variables*: 2, 10, 13; *Interval-scaled variables:* 8,9,15,16; *dichotomous variables:* Dep. Variable, 1 (0 = breast and bowel centres; 1 = Referred if needed), 3 (0 = men; 1 = women), 4,5,6,7, 14 (0 = no; 1 = given). Other dichotomous variables: not applicable (0), applicable (1). Bold figures: significant differences (p < 0.05).

## Discussion and conclusions

The principal focus of this study was the assessment of psychological distress rather than of major psychiatric disorders. Patients selectively referred because of suspected psychosocial comorbidity showed more physical and psychological distress and a higher burden; similar tendencies were found for patients suffering from bowel cancer in comparison with those suffering from breast cancer. Mood swings, anxiety, depressed mood or grief, and fatigue were the most important distress symptoms. Fatigue, confirmed metastases, and functional limitations in daily activities may predict a higher level of psychological distress. Referral mode, gender, family problems, fatigue, and previous psychiatric treatment may predict the need for further psychosocial support after discharge, whereas in cases of increasing age, recommendations for outpatient psychosocial support decrease.

The predictors of psychological distress and of indication for further psychosocial support found in this investigation are in agreement with most of the published research. Advanced stage of disease, even more when associated with functional impairment and high symptom burden, was repeatedly found to predict psychosocial distress and co-morbidity.

Many psycho-oncological investigations have dealt with the prevalence of psychiatric disorders, showing disparate results. Singer et al. found, on the basis of a meta-analysis of 1,448 cases, that one-third of those in acute care hospitals suffered from mental health disorders [18]. Härter et al. found prevalence rates of 23.5% for mental disorders among patients in the last 4 weeks [1]. Kadan-Lottik et al., in a cross-sectional, multi-institutional study of patients with advanced cancer, found that 12% met the criteria for a major psychiatric condition [19]. Brinttzenhofe-Szoc et al. demonstrated for a large sample of n = 8,265 patients assessed by means of the Brief Symptom Inventory that 70% did not meet thresholds for depression or anxiety symptoms; it can be interpreted as a reflection of the resistance to developing a significant level of these symptoms [20]. When psychological distress was assessed in terms of relatively minor disorders, the share of impaired patients increased. So, 37.8% of a large sample (n = 2,776) assessed by means of Brief Symptom Inventory (BSI)-18 over a 4-week period met the criteria for general distress in the clinical range [21]. Zabora et al. found, in another large sample (n = 4,496), a similar rate of 35.2% [22]. Mehnert and Koch found, for a sample consisting of 1,083 cancer patients over a 47-month period, prevalence rates for psychological distress of about 43% (minor psychiatric disorders) and 26% (major psychiatric disorders); the most frequent problems were anxiety (38%), depression (22%), and posttraumatic stress disorder (12%) [23]. Psychological distress seems to be more frequent in inpatient than in outpatient care [24] and in palliative care settings than in other inpatient settings [25].

Overall, the prevalence rates found in this study for psychiatric disorders (6%), and for major psychological distress, corresponding to distress scores for the preceding 3 days of “considerable” or “very much” (20% for depression, 21% for anxiety, and 19% for mood swings), were lower than those found in other recent investigations. Possible reasons for this include use of different classification manuals (ICD-10 vs. DSM-IV), differences in setting, different survey time frames, confusion of major and minor psychological and psychiatric disorders and stresses, raters’ different clinical experiences as well as different expectations, and differences in patients’ or experts’ perspectives.

Results of this investigation are similar to those of Siedentopf et al. [26] for 333 breast cancer patients also assessed by PO-Bado. Fatigue was scored the highest in the physical distress dimension, anxiety and depression in the psychological dimension. The proportion of patients who needed psychosocial support was similar (23% vs. 30%), but we found more predictors for need for further professional support after discharge. Discrepancies to other authors’ results may result from younger age, where cancer is known to affect many social roles as parents, as earners of the family’s living etc. [27,28] possibly causing less conflicting strain when cancer occurs at older age. So, our results demonstrate a decreasing advice for further professional psychosocial support after discharge related to increasing age, contrary to the results of Akechi et al. [29]. We did not find a higher prevalence associated with unemployment, as did Singer et al. [30], or with the presence of underage children in the family, as did Ernst et al. [31].

Professionals’ and patients’ views often differ on the need for further psychosocial support [32]. In a Belgian survey, one female cancer patient out of four and one male patient out of ten desired psychological support [33]. This difference raises the question of met and unmet needs in cancer patients. In a retrospective study administrated by Erstmann et al. to 710 German cancer patients, 18.9%, especially women, indicated unmet needs for psychosocial support, and 9.5% were actually using psychosocial services [34]. An English survey on the basis of a psychosocial needs inventory comprising seven needs categories demonstrated that psychosocial needs were commonly expressed and strongly felt by cancer patients; particular needs were related to tumor type, illness critical moments, age, gender, health status, socio-economic status, and other factors [35]. In an Australian study on the basis of a small sample, strength of unmet needs was associated with anxiety, depression, posttraumatic stress, poorer quality of life, younger age, and greater time since diagnosis [36]. Our investigation was focused on expert opinions about need for further psychosocial support; it confirmed the importance of gender, age, presence of metastases, fatigue, previous psychiatric or psychotherapeutic treatment, and family problems, but not pain, socio-economic problems, duration of illness, or limitations in functional capacity-perhaps because of good medical care and ambulatory nurse support as well as protective relationships in rural areas and the presence of fewer economic problems in the surveyed region. The authors found a discrepancy of predictors for indication of further psychosocial support as partially opposed to associations to psychological distress. Here, hardly illness-related factors are found, but lack of adequate social support, due to family problems among mostly female patients. Perhaps that health professionals’ indication is colored by subjective impressions of women in need, who would otherwise not call for professional support. Moreover, health professionals’ estimate of patients in need for support also entails patients’ motivation and an anticipated benefit of such psychosocial support. However, the fact that selectively referred patients because of suspected psychosocial comorbidity -who really show more psychological strains- triggers significant more indications for further psychosocial treatment indicates some decision accuracy on the part of CLS professionals.

As Carlson et al. suggested, screening may improve communication between patients and clinicians and may enhance psychosocial referrals, but its direct effects on quality of life are uncertain [37]. On the other hand, Zabora et al. argue that early identification of distress with appropriate interventions could reduce distress, enhance quality of life, and decrease health care costs [38]. In summary, we agree to Mitchell et al. that both over- and under-detection of psychological distress are problematic and that screening is generally ineffective without aftercare [39].

## Abbreviations

CLS: Consultation-liaison service; OR: Odds ratio; PO-Bado: Psycho-Oncological Basic Documentation; VIF: Variance inflation factor.

## Competing interests

The authors declare that they have no competing interests.

## Author’s contributions

JVS as corresponding author conceived of the study, performed the statistical analyses, and drafted the manuscript. EV participated as consultation-liaison therapist, collected the data and participated in the design of the study. RK participated in the design of the study, assessed and supervised the statistics, and participated in the draft of the study. All authors read and approved the final manuscript.

## Pre-publication history

The pre-publication history for this paper can be accessed here:

http://www.biomedcentral.com/1471-244X/13/226/prepub
